# Working in partnership with communities to improve health and research outcomes. Comparisons and commonalities between the UK and South Africa

**DOI:** 10.1017/S1463423619000677

**Published:** 2019-09-10

**Authors:** Patricia Wilson, Azwihangwisi Helen Mavhandu-Mudzusi

**Affiliations:** 1 Professor, Centre for Health Services Studies, University of Kent, Canterbury, UK; 2 Chair, Department of Health Studies, University of South Africa, Pretoria, South Africa

**Keywords:** community engagement, community health, community participation, patient and public involvement, primary care research

## Abstract

Community and public participation and involvement is an underpinning principle of primary health care, an essential component of a social justice-orientated approach to health care and a vehicle to improving health outcomes for patients, public and communities. However, influenced by history and context, there are intrinsic issues surrounding power imbalance and other barriers to partnerships between communities, public, policy makers and researchers. It is important to acknowledge these issues, and through doing so share experiences and learn from those working within very different settings.

In South Africa, community participation is seen as a route to decolonisation. It is also integral to the core functions of South African Higher Education Institutes, alongside teaching and research. In the UK, there has also been a history of participation and involvement as part of a social rights movement, but notably public involvement has become embedded in publicly funded health research as a policy imperative.

In this paper, we draw on our respective programmes of work in public and community participation and involvement. These include a South African community engagement project to reduce teenage pregnancy and HIV infection working through a partnership between teachers, students and university academics, and a national evaluation in England of public involvement in applied health research. We begin by highlighting the lack of clarity and terms used interchangeably to describe participation, engagement and involvement. Frameworks for partnership working with relevance to South Africa and the UK are then analysed, suggesting key themes of relationships, working together, and evaluation and monitoring. The South African project and examples of public involvement in English primary and community care research are examined through these themes. We conclude the paper by mapping out common enablers and barriers to partnership working within these very different contexts.

## Introduction

Enabling community and public participation is an underpinning principle of primary health care and enshrined within the Alma Ata Declaration (World Health Organization, 1978). Community and public participation is seen as emblematic of postmodern democracies, a response to health care decisions failing to include the perspectives and views of the recipients (Entwistle *et al.*, 1998). In lower- and middle-income countries, community and public participation is also seen as an essential element of a human rights-based approach (Marston *et al.*, 2016) and as a process that guards against exploitation (Lavery *et al.*, 2010). In countries such as South Africa, community and public participation is viewed as a way to decolonisation which is a process of obtaining social justice (Israel *et al.*, 2012). However, this social justice-orientated approach to participation is just one rationale. An additional justification for community and public participation emerges from a consumerist (Stewart, 2013) or utilitarian perspective (Brunton *et al.*, 2017). This argues that participation can lead to improved health outcomes (O’Mara-Eves *et al.*, 2015; National Institute for Health and Care Excellence, 2016), with examples including improving maternal and neonatal health in Bangladesh (Marcil *et al.*, 2016) and managing epidemics such as Ebola (Laverack and Manoncourt, 2016). Yet, these sometimes competing rationales have led to potential confusion and ‘terminological instability’ (Stewart, 2013), illustrated by terms such as participation, involvement, engagement and co-production being used interchangeably. This inconsistency is further compounded by differences in how those involved are defined including: how communities are demarcated; differences between public, citizens, consumers, stakeholders and users and levels of participation ranging from passive consultation to community-led initiatives (Arnstein, 1969).

These different justifications and heterogeneous dynamics are mirrored in community and public participation in health-related research. Legitimacy has been framed by the ethical argument that individuals and communities have a right to be fully involved particularly when research interventions are being done ‘to’ them (Barnes and Cotterell, 2012). Furthermore, the right to be involved developed further with a range of emancipatory approaches leading to user- or community-led research (Oliver, 1992). Alongside this argument, the justification that community and public participation leads to more relevant research with a greater chance of being implemented has resulted in public participation being increasingly embedded within policy and guidelines (Wilson *et al.*, 2018). Examples include the requirements for patient and public involvement in the UK publicly funded health research (Staniszewska *et al.*, 2018) and the incorporation of community engagement within South Africa’s National Health Research Ethics Council’s guidelines for good practice in clinical research (MacQueen *et al.*, 2015).

The objective of this paper is to provide a cross-cultural analysis of the enablers and barriers to community participation and involvement in health care and research. The paper is based on the assumption that community and public participation is of inherent value and an ethical prerequisite despite lack of conceptual clarity and differences in contextual setting. However, we also acknowledge intrinsic issues surrounding power imbalance and other barriers to partnership between communities, public, policy makers and researchers (Brunger and Wall, 2016; Marston *et al.*, 2016; McCollum *et al.*, 2018). We explore similarities and differences in frequently used terms and develop a set of underlying principles to underpin our discussion. Drawing on our respective programmes of work, we compare and contrast approaches to community and public participation in primary and community care research and projects. While the work we describe is very different, we conclude the paper with key transferable lessons between the UK and South African primary care and community research settings.

## Definitions

The plethora of terms used in this field not only reflects how value-laden it is (Stewart, 2013) but also regional differences and nuances in language. For example, in North America, the Canadian Institutes of Health Research’s Strategy for Patient-Oriented Research and the US Patient-Centered Outcomes Research Institute use the term engagement to describe the partnership approach aimed at enabling patient-identified priorities for research (Chudyk *et al.*, 2018). In the UK, public involvement in research is the predominate semantic, in which the National Institute for Health Research (NIHR) national advisory group for public involvement (INVOLVE) defines as ‘research being carried out “with” or “by” members of the public rather than “to”, “about” or “for” them’ (Hayes *et al.*, 2012). In contrast, the UK understanding of engagement is limited to the provision and dissemination of information about research (https://www.invo.org.uk/resource-centre/jargon-buster/?letterE). In South African academic institutions, community and public participation is understood as community engagement, which is considered as one of the core functions of universities alongside teaching and research (Lazarus *et al.*, 2008).

However, community engagement is similarly poorly defined (Odugleh-Kolev and Parrish-Sprowl, 2018) and known by a number of terms including community participation (Preston *et al.*, 2010; George *et al.*, 2015). Tindana *et al.* (2007) suggest that community engagement in research goes beyond community participation; it is a process that requires collaborative working with partners who share goals and interests. While there is some terminological blurring with the World Health Organization’s concept of community development (Kang *et al.*, 2016), at a World Health Organization (2017) workshop aimed at developing a community engagement framework, it was defined as a ‘process of developing relationships that enable stakeholders to work together to address health-related issues and promote well-being to achieve positive health impact and outcomes’. Definitional fluidity is perhaps inevitable as there is no standard definition of community (Tindana *et al.*, 2007). It may commonly demarcate a geographic area (eg, a remote rural community), but could also be used to characterise a group of people living with a particular health condition or a group sharing particular value systems and cultural characteristics (George *et al.*, 2015; Kang *et al.*, 2016). In the South African health setting, community participation is mainly related to primary health care nurses and community health workers (currently called Ward-based Primary Health Care outreach team), who go out to the community or to the homes of community members to offer health-related information and care (Kironder and Kahirimbanyi, 2002). Other terms commonly used in South Africa include community outreach and community mobile teams.

Despite the difficulties in pinning down definitions, it is perhaps the level of participation, involvement or engagement (or whatever local term is used) that characterises differences in values and approaches most graphically. A number of conceptual frameworks have been developed to describe the spectrum of community and public involvement, for example, the IAP2 Spectrum of Public Participation (International Association of Public Participation, 2018) and Taylor *et al.*’s (2008) four conceptual approaches to community participation. These are described as ‘contributions’ where external stakeholders facilitate and use contributions from the community; ‘instrumental’ with community participation as part of an intervention to improve health; ‘community empowerment’ underpinned by social action and ‘developmental’ as an evolutionary partnership process based on social justice. Most of these frameworks have roots and similarities to the seminal work of Arnstein (1969) who describes the range of levels as a ladder from non-participation/manipulation to ‘citizen-led control’. More recent work has questioned the linear nature of this ladder model, suggesting that different levels may occur at different stages of a research project (Wilson *et al.*, 2015). However, the concept of levels of involvement or participation is a useful one and if critically explored can unmask assumptions and covert power imbalance (Brunger and Wall, 2016).

In England, this critique was demonstrated in a commissioned review of public involvement in research (National Institute of Health Research, 2015). This recommended that co-production should provide the principles for the future NIHR approach to public involvement in research (Staniszewska *et al.*, 2018). Co-production is yet another term that is used interchangeably with others such as co-design and is conceptualised in nuanced ways. While co-design normally refers to collaborative planning and designing of health care services (and research) by patients/service users and clinicians/managers, co-production is an umbrella term denoting joint working between these groups and includes policy making, governance, design and delivery of care (Prior and Campbell, 2018; Baim-Lance *et al.*). The NIHR drew on the work of Boyle *et al.* (2010) to develop guidelines for co-production of health research (INVOLVE, 2018). What clearly demarcates this approach to research is the overt sharing of power and joint ownership of the research by the public involved and the researchers (INVOLVE, 2018). While it is acknowledged that co-produced research may not be possible in all circumstances, it does demonstrate an intentioned shift ‘up the ladder’ of involvement.

## Underpinning principles

We build on an earlier literature review conducted by one of the authors as part of the RAPPORT study, conducted 2012–2015 in England. Funded by the NIHR, the study sought to evaluate different approaches, impact and outcomes of partnership working within publicly funded health research. In particular, it sought to assess the mechanisms that lead to partnership working being routinely incorporated in the research process. The methodology and findings are reported elsewhere (Mathie *et al.*, 2014; Wilson *et al.*, 2015; Howe *et al.*, 2017; Wilson *et al.*, 2018), but in summary a realist design was used (Pawson, 2013), which drew upon Normalization Process Theory (May *et al.*, 2018) to understand how partnership working became embedded as normal practice. The review was updated, and grey literature included through searches on Open Grey. Our purpose was to identify exemplar frameworks that could illuminate practices relevant to the UK and South Africa health care and research environments. We included papers and reports on community engagement or participation, public involvement in research or co-production of health research that were:

- Published in English from 2009 onwards
- Included frameworks or best practice guidelines applicable to the UK and South Africa.

In Table 1, we summarise the main principles from the exemplar frameworks and best practice guidelines.

**Table 1.** Examples of frameworks and best practice guidelines for public involvement, community engagement and co-production of health and health research

	Type	Source and purpose	Main principles
Wilson *et al.* (2015)	Public involvement in research	National evaluation of PI in publicly funded health research in England. Identified six salient actions to embed PI as normal practice	A clear purpose, role and structure for PI are ensured within the research The research team actively recruits lay representatives who are likely to have an understanding of the diverse viewpoints of the study’s target population All researchers within the team engage with PI, acknowledge the need to explain technical aspects of the research and have the skills to do so Researchers and lay representatives acknowledge, understand and trust each other’s contributions Opportunities for PI in all parts of the research process are fully exploited Researchers and lay representatives participate in ongoing reflections and evaluate PI in order to improve the quality of PI processes and outcomes
National Institute for Health Research (2017)	Public involvement in research	Stakeholder development of national standards for public involvement in research in England	Clear, meaningful and accessible opportunities for involvement, for a wide range of people across all research Create and sustain respectful relationships, policies, practices and environments for effective working in research Public involvement is undertaken with confidence and competence by everyone through adequate support and learning Clear and regular communications are provided as part of all involvement plans and activities The impact of involving the public in research is assessed, reported and acted upon Ensure the community of interest voices are heard, valued and included in decision-making.
National Institute for Health and Care Excellence (2016)	Community engagement	Principles of good practice and guidelines for community engagement in the UK. Main focus public health	Ensure local communities, community and voluntary sector organisations and statutory services work together to plan, design, develop, deliver and evaluate health and well-being initiatives Recognise that building relationships, trust, commitment, leadership and capacity across local communities and statutory organisations needs time Support and promote sustainable community engagement by encouraging local communities to get involved in all stages of a health and well-being initiative Ensure decision-making groups include members of the local community who reflect the diversity of that community. Encourage individual members to share the views of their wider networks and others in the community Feedback the results of engagement to the local communities concerned, as well as other partners.
Lavery *et al.* (2010)	Community engagement	Development of a framework for broader discussions of community engagement in global health research.	Rigorous site selection procedures Early initiation of community engagement activities Characterise and build knowledge of the community, its diversity and its changing needs Ensure the purpose and goals of the research are clear to the community Provide information Establish relationships and commitments to build trust with relevant authorities in the community: formal, informal and traditional Understand community perceptions and attitudes about the proposed research Identify, mobilise and develop relevant community assets and capacity Maximise opportunities for stewardship, ownership and shared control by the community Ensure adequate opportunities and respect for dissenting opinions Secure permission/authorisation from the community Review, evaluate and if necessary, modify engagement strategies
World Health Organization (2017)	Community engagement	Development of a framework for community engagement through consensus workshops	Governance structures and process for CE should be informed by regional/local contextual environment It requires leadership, clear strategic priorities and goals Resources are not limited to financial, equipment, etc, but include time, spaces, technology and communication skills to support participatory processes and deep listening to connect with authenticity The health workforce should receive the training to engage in dialogue and develop partnerships with the community. Requires robust monitoring and evaluation to enable successful design and implementation
Boyle & Harris (2009)	Co-production	Development of defining characteristics of co-production to inform how public services are conceptualised, designed and delivered in the UK.	Recognising people as assets: transforming the perception of people from passive recipients of services and burdens on the system into one where they are equal partners in designing and delivering services. Building on people’s existing capabilities: altering the delivery model of public services from a deficit approach to one that provides opportunities to recognise and grow people’s capabilities and actively support them to put these to use with individuals and communities. Mutuality and reciprocity: offering people a range of incentives to engage, which enable us to work in reciprocal relationships with professionals and with each other, where there are mutual responsibilities and expectations. Peer support networks: engaging peer and personal networks alongside professionals as the best way of transferring knowledge and supporting change. Blurring distinctions: blurring the distinction between professionals and recipients, and between producers and consumers of services, by reconfiguring the way services are developed and delivered. Facilitating rather than delivering: enabling public service agencies to become catalysts and facilitators of change rather than central providers of services themselves.
INVOLVE (2018)	Co-production	Stakeholder development of guidelines for the co-production of health research	Sharing of power – the research is jointly owned and people work together to achieve a joint understanding Including all perspectives and skills – make sure the research team includes all those who can make a contribution Respecting and valuing the knowledge of all those working together on the research – everyone is of equal importance Reciprocity – everybody benefits from working together Building and maintaining relationships – an emphasis on relationships is key to sharing power. There needs to be joint understanding and consensus and clarity over roles and responsibilities. It is also important to value people and unlock their potential.

Abbreviations: PI – public involvement, CE – community engagement.

An analysis of these principles suggests three main themes – relationships, working together and evaluation and monitoring.

### Relationships

Developing and sustaining relationships based on trust are commonly described as the foundation for public involvement and community engagement. These relationships are characterised by mutual respect (Wilson *et al.*, 2015; National Institute for Health Research, 2017) and include the need to understand the community’s perspective (Lavery *et al.*, 2010). Relationships also need time to develop (Wilson *et al.*, 2015; National Institute for Health and Care Excellence, 2016), and as such involvement and engagement needs to be started as early as possible in the project or initiative (Lavery *et al.*, 2010; Wilson *et al.*, 2015; National Institute for Health and Care Excellence, 2016). Authentic relationships require accessible and inclusive opportunities (National Institute for Health Research, 2017) to ensure that public and community members are those most likely to be able to speak for and link with their peers (Lavery *et al.*, 2010; Wilson *et al.*, 2015; National Institute for Health and Care Excellence, 2016). Developing authentic relationships also requires specific communication skills on the part of the external stakeholder (National Institute for Health Research, 2017; World Health Organization, 2017). In the UK research environment, these skills may be seen as the remit of one person within a research team, but the evidence suggests that for involvement to be embedded as normal practice, all research members need to be part of the partnership with involved public and patients (Wilson *et al.*, 2018). Clear communication and providing feedback between the relevant parties are identified in a number of the guidelines and frameworks (Lavery *et al.*, 2010; Wilson *et al.*, 2015; National Institute for Health and Care Excellence, 2016; National Institute for Health Research, 2017). Trust is also facilitated by sharing a clear purpose and goals for public involvement and community engagement (Lavery *et al.*, 2010; Wilson *et al.*, 2015; World Health Organization, 2017). However, a lack of clarity around the relationship affects participation, often rendering the community as passive recipients who have very little to no say in the process of participation and what they need (Brunton *et al.*, 2017). The literature on community participation commonly highlights the working relationship as an issue. The community acts as observers, while health care practitioners take the leading role. Most of the time, the community members’ roles would only be to invite the people to the venue, while the primary health care workers conduct all the key functioning related to partnership, including giving instructions to community members, negatively impacting the sustainability of the partnership (Wallerstein and Duran, 2006).

### Working together

Closely linked to sustainable relationships are the ways researchers, public, external stakeholders and communities work together. There is a strong theme of reciprocity, a recognition of equal value of all perspectives (National Institute for Health Research, 2017) and adopting an assets-based approach (Boyle *et al.*, 2010; Lavery *et al.*, 2010). The way of working is also influenced by the level of public involvement and community engagement. In the latter, community empowerment approaches implicitly shift the balance of power to the community (Taylor *et al.*, 2008), and in research, co-production requires a sharing of power through the joint ownership of research (Lavery *et al.*, 2010; INVOLVE, 2018).

In multi-ethnic, multilingual countries such as South Africa, it is sometimes challenging when people who are engaged in community engagement are from a different ethnicity with different cultural values and beliefs. This often leads to misunderstanding and, instead of bringing positive outcomes, can result in a negative impact on the project.

### Evaluation and monitoring

Several guidelines and frameworks highlight the need for ongoing evaluation and monitoring of public involvement and community engagement if best processes and outcomes are to be achieved (Lavery *et al.*, 2010; Wilson *et al.*, 2015; National Institute for Health Research, 2017; World Health Organization, 2017). However, this is not without challenge as there are methodological difficulties in assessing the impact of public involvement in research (Wilson *et al.*, 2015) and community engagement (Preston *et al.*, 2010). From the social justice perspective, there is also some ongoing debate whether it is appropriate to evaluate the ‘moral right’ to be involved as an intervention (Wilson *et al.*, 2018). In reality, the processes and outcomes of most partnership working are not known. Commonly, this is because community partnership becomes just a once-off event or only conducted to meet the organisational needs or plans. It becomes like a tickbox action on the part of the initiating institution or department without any follow-up of the impact as monitoring and evaluation are not built into the project plan (Szilagyi *et al.*, 2014).

Relationships and ways of working demonstrate some level of partnership, and from herein, we use the term ‘partnership working’ to describe public involvement in research, and community participation and engagement. Henceforth, we also use the term ‘public’ or ‘community partners’. We now provide examples of partnership working from the English primary and community research context and community partnership with schools in South Africa.

## Partnership working in English health research: examples from a national evaluation

Within the RAPPORT study, 22 studies were followed over 18 months to track partnership working processes and outcomes (Wilson *et al.*, 2015). Examples of three studies based in the primary or community settings are now presented, with an analysis of their relative strengths and weaknesses through the lenses of relationships, working together and evaluation. Data were collected through semi-structured interviews and document review using a realist approach (Marchal *et al.*, 2010; Manzano, 2016). Interviews were digitally recorded, transcribed and analysed using a framework approach (Gale *et al.*, 2013). A breakdown of participants is presented in Table 2.

**Table 2.** Study participants

Study identification	Number of public partners interviewed	Number of researchers interviewed	Total number of interviews conducted over 18 months
1	2	3	6
2	1	3	5
3	2	5	7

### Study 1: Trial of self-help materials for the prevention of smoking relapse: randomised controlled trial with parallel qualitative study

Two public partners were part of the trial steering group, with a third partner consulted on the intervention booklets. In addition, a local established public partners group advised on participant information materials submitted to the relevant research ethics committee. Impact from all their involvement included refinement of the self-help booklets to be better tailored for the target population and improved clarity of participant materials.

In terms of relationships and working together, the lead researcher had little experience in working in partnership with the public. Their main motive for partnership working was the requirement from the funder; in the UK, publicly funded applied health research grants will not be awarded without partnership working. The lead researcher was not convinced of the value of partnership working, perceiving there to be a lack of evidence of positive impact on research:

*…researchers want evidence (on value of partnership working) and that’s the holy grail, and I haven’t seen it yet…*

The lead researcher also questioned the costs associated with partnership working, including time and fees to public partners for their contributions. This rather negative view resulted in superficial levels of partnership working, mainly through one-off consultations. This led to disengagement by public partners who removed themselves from the study.

### Study 2: Physical activity in older people in primary care: randomised controlled trial

This study had been preceded by a pilot study, and participants from the pilot provided input into the development of intervention through a series of focus groups. Another group of public partners was bought together as a focus group to contribute to participant information before submission to the ethics committee. Throughout the study, a public partner from a local community voluntary service was a member of the study advisory group. Partnership working impact on this study included a change in the images and terminology on participant information (public partners had raised concerns about potential ‘ageism’). The lead researcher also attributed considerable changes to the study design to partnership working.

This study demonstrated the blurring of boundaries between qualitative research (focus groups) and partnership working. This has been highlighted as a potential issue if the purpose is not clear (Wilson *et al.*, 2015). If data are being collected through qualitative methods, this is clearly research rather than partnership working, even if it has potential to contribute to partnership working through enabling the public and community perspective to be explored (Rolfe *et al.*, 2018). Many of the researchers in this team saw working together interchangeably as partnership working and study participation. However, the public partner member of the study advisory group who was involved solely in the project governance saw her contribution to partnership working as a service to her local community and fundamental to research:

*I think it’s (partnership working) fundamental to the research inquiry…it’s not just added value, it’s intrinsic to the inquiry*.

There was demonstrable positive impact on the study through partnership working. One of the strong features was the relationships between the lead researcher, research nurse and public partners. The research nurse had been the main contact with the preceding pilot study participants, which facilitated the participation of 60 people in the ‘trial design development’ focus groups. As way of evaluation and monitoring, the research team recorded all partnership working contributions and subsequent changes. However, these changes were not systematically fed back to public partners.

### Study 3: Evaluation of peer-led interventions for parents of children with intellectual and developmental disabilities: a systematic review and qualitative study

This study was based in an established research centre that continuously worked with a large group of parents as public partners. Working together was characterised by co-production. The research idea had come from the parents, and a subgroup was formed of parents and researchers to jointly work together at each stage of the project. The impact of this partnership working included changing the study design, improving recruitment to the qualitative study, increased relevance of outcome measures, parents setting the inclusion criteria for the review and dissemination of findings. This study also had a significant impact on the public partners themselves, reporting increased self-worth and knowledge of health services and providing a respite from the daily care of their child.

A ‘family coordinator’, who the lead researcher described as being the bridge between the research and parent worlds, facilitated the relationship between the public partners and researchers:

*…she knows enough about research to be able to explain what we do, but equally doesn’t know enough that she can challenge me or others in the team to explain more clearly what we’re asking of families. And I think that’s probably the greatest reason for our success has been the sort of creation of that role and the development of it*

However, while the coordinator dealt with the day-to-day work with parents, the whole research team was committed to partnership working and many had applied to work at the centre because of the long-established co-production model.

In addition to this sustained relationship maintained over time through working together and regular social events, there was reimbursement for parents’ time and a training and support programme. However, this level of partnership working required not only skills and commitment but also financial resources, which many other studies within the RAPPORT evaluation lacked. These financial resources also freed up staff time to conduct evaluation and ensured feedback to public partners on how their input had impact.

## Partnership working in South Africa: a community engagement project with schools

Enhancing HIV prevention among learners in Limpopo was a collaborative project between the members of the Department of Health Studies at the University of South Africa and the schools in the Soutpansberg North Circuit. The background to the project was that learners at rural schools continued to be infected with HIV despite the currently offered HIV/AIDS Life Orientation programme at schools, with the programme failing to address the social, economic and cultural context of these learners. The community engagement project focused on developing and translating an HIV/AIDS education toolkit into the local languages used by learners. Teachers together with academics developed the initial content. However, the final content was co-designed by academics, educators and learners who were actively involved during the pilot phase of the toolkit. The language used was that of the learners; the common parlance from their daily lives and aspects addressed included focusing on what learners’ experience daily. Roles were agreed as the teacher being just a facilitator, while learners led the discussion and demonstrations, for example, demonstration of condom use and also disposal. This resulted in mutual respect and taking ownership. Due to the reciprocity in the relationship, participants, especially learners, were able to share their experiences, challenges and also factors which contributed to their engagement in HIV risky sexual behaviour. This helped to identify contextual data and the planning of a relevant intervention which was acceptable to all participants. All the stakeholders kept to their roles, ensuring that the programme’s objectives were achieved. For example, the academics were fully involved in fundraising to provide learners with school uniforms, sanitary towels and clothes. This was in response to learners suggesting that they became sexually active because of the lack of these items. The relationship developed between all stakeholders resulted in a commitment by learners to change their sexual behaviour and improved their school attendance. The project also influenced educators to continue to use participatory approaches within HIV/AIDS education. When the project started in 2014, out of 183 learners aged 6–18 years, there were 25 learners who were pregnant. This had been a trend for a number of years, and it had become an expected norm that girls above 12 years old were pregnant. However, two years after the project commenced, there were no pregnant learners at school and this trend has been sustained up to 2018. Policy makers are now taking note of the programme, and it is being rolled out to other schools.

## Enablers and challenges in partnership working

Our respective partnership working has been conducted in significantly different settings, with large variation in resources available, public and community partners involved, and overall aims of the projects. However, all the work we have described can be looked through the social justice and utilitarian lenses, giving voice and a participatory role to those involved in the projects and harnessing this voice and role to improve health outcomes. The examples of partnership working across such a broad landscape also reveal a number of common enablers and barriers.

### Enablers

#### Mutual benefit

Both partners should be able to benefit from the project. For example, in the project on HIV prevention among learners in Limpopo Province, the community, especially the teachers, benefited from the project. The project enabled them to teach learners contextually relevant HIV prevention messages and approaches, leading to a reduction in the risk of HIV infection among learners. Healthy learners attend schools more regularly, and their performance improves. This positively affects the pass rate, bringing esteem to the teacher, the school and the community. The learners who have performed well are able to access the university which is a partner in the project. Students who have good attainment from school perform better, improving the university ratings, which contributes to sustained funding. This enables the university to fully commit to community engagement projects thus forming a cycle of mutual benefit.

This virtuous cycle of mutual benefit was also a finding in the RAPPORT study. While resource-intensive, working in partnership led to improved research prioritisation and identification of questions, design and research outcomes. It also enhanced self-esteem of public partners and the researchers. Working together became cyclical, with those involved developing ideas and design of future projects based on their experiences of the one they were currently involved in (Wilson *et al*., 2015).

Mutual benefit is also enhanced if the project has relevancy for all those involved. In the Limpopo Province HIV prevention among learners project, the relevance of the work was clearly identifiable. However, in the RAPPORT study, there were some studies where public partners questioned the relevance for them. Nevertheless, partnership working could address relevancy. For example, the outcome measures used within a study on rheumatoid arthritis were questioned by public partners and more patient-relevant measures were introduced. The relevance of the project needs to be identified at the start to ensure a receptive environment and ‘buy-in’ of those involved, but equally as the project progresses (and as shown by the South African example) there should be an iterative and flexible approach to ensure the project is adapted to maintain relevance.

#### Resources

Partnership working is resource-intensive, not least in the time and commitment required by all involved. This commitment was demonstrated in the South African example with academics joining in fundraising events. Similar examples were also found in the RAPPORT study, with academics fundraising for charities to enable grant awards. In the RAPPORT study, there were examples of very well-resourced research centres that had sustained funding for partnership working leads and activities. However, there were other examples where there was little to no funding for partnership working, and meaningful working together relied on commitment of all those involved, and most importantly on trusting relationships. While not ideal, this is often the reality, particularly when starting out in partnership working. However, as described in the HIV prevention among learners in Limpopo Province project, mutual benefit for all those involved leads to sustained support from partners to ensure that the objectives of partnership are achieved. In the RAPPORT study, we also found that sustained support through trusting relationships was further enabled through mutual agreement of the roles of all involved and the purpose of partnership working.

### Barriers

#### Limited time

In both the South African and UK examples, time was one of the most precious resources required for partnership working. In RAPPORT case studies, time was needed to develop initial relationships with public partners, often during the pre-grant award period. Time was also needed to maintain these relationships throughout the project period and beyond. Paradoxically, this time-intensive work was often needed most when the research team was most constrained in time, working towards a grant application deadline or during the busy project set-up period or writing up. In the HIV prevention among learners in Limpopo Province project, competing demands on time included delivering and learning the rest of the curriculum for teachers and learners, and working on the project among other university commitments for the academics. Participatory approaches within projects are also time-intensive including the organising and running of workshops and integrating their outcomes into the project. Commitment and careful planning are required to overcome this barrier.

#### Sudden attrition of partners especially the vision holders

To embed what may be a new way of working to those involved requires a champion or vision holder. The RAPPORT study found a number of these key people, including lead researchers, partnership working coordinators or members of patient organisations. In teams where this vision of partnership working was held by all, then the approach was sustained and well embedded throughout all activities. However, where reliant on one person within a team, any loss of this role could result in the demise of partnership working into one that was tokenistic at best.

## Conclusion

We have explored the differing definitions and environments within which partnership working can work. While there were differences in the purpose of the projects we described, the basic principles apply to them all. The examples presented were all underpinned by a rationale of social justice and utilitarianism, but shaped by history and culture. Nevertheless, we found commonalities in enablers and barriers to partnership working, and the potential for mutual learning. Partnership working in LMICs such as South Africa provides exemplars of innovative participatory approaches firmly shaped within the social justice paradigm, but with utilitarian outcomes for community health. Our experiences of partnership working in England can also provide lessons on what needs to be done to embed such practices as policy (Wilson *et al*., 2018).
